# Dynamic arterial elastance as a predictor of arterial pressure response to fluid administration: a validation study

**DOI:** 10.1186/s13054-014-0626-6

**Published:** 2014-11-19

**Authors:** Manuel Ignacio Monge García, Manuel Gracia Romero, Anselmo Gil Cano, Hollmann D Aya, Andrew Rhodes, Robert Michael Grounds, Maurizio Cecconi

**Affiliations:** Servicio de Cuidados Intensivos y Urgencias, Hospital SAS de Jerez, C/Circunvalación s/n, 11407 Jerez de la Frontera, Spain; Department of Intensive Care Medicine, St George’s Healthcare NHS Trust and St George’s University of London, Blackshaw Road, Tooting, London, SW17 0QT UK

## Abstract

**Introduction:**

Functional assessment of arterial load by dynamic arterial elastance (Ea_dyn_), defined as the ratio between pulse pressure variation (PPV) and stroke volume variation (SVV), has recently been shown to predict the arterial pressure response to volume expansion (VE) in hypotensive, preload-dependent patients. However, because both SVV and PPV were obtained from pulse pressure analysis, a mathematical coupling factor could not be excluded. We therefore designed this study to confirm whether Ea_dyn_, obtained from two independent signals, allows the prediction of arterial pressure response to VE in fluid-responsive patients.

**Methods:**

We analyzed the response of arterial pressure to an intravenous infusion of 500 ml of normal saline in 53 mechanically ventilated patients with acute circulatory failure and preserved preload dependence. Ea_dyn_ was calculated as the simultaneous ratio between PPV (obtained from an arterial line) and SVV (obtained by esophageal Doppler imaging). A total of 80 fluid challenges were performed (median, 1.5 per patient; interquartile range, 1 to 2). Patients were classified according to the increase in mean arterial pressure (MAP) after fluid administration in pressure responders (≥10%) and non-responders.

**Results:**

Thirty-three fluid challenges (41.2%) significantly increased MAP. At baseline, Ea_dyn_ was higher in pressure responders (1.04 ± 0.28 versus 0.60 ± 0.14; *P* <0.0001). Preinfusion Ea_dyn_ was related to changes in MAP after fluid administration (*R*^2^ = 0.60; *P* <0.0001). At baseline, Ea_dyn_ predicted the arterial pressure increase to volume expansion (area under the receiver operating characteristic curve, 0.94; 95% confidence interval (CI): 0.86 to 0.98; *P* <0.0001). A preinfusion Ea_dyn_ value ≥0.73 (gray zone: 0.72 to 0.88) discriminated pressure responder patients with a sensitivity of 90.9% (95% CI: 75.6 to 98.1%) and a specificity of 91.5% (95% CI: 79.6 to 97.6%).

**Conclusions:**

Functional assessment of arterial load by Ea_dyn_, obtained from two independent signals, enabled the prediction of arterial pressure response to fluid administration in mechanically ventilated, preload-dependent patients with acute circulatory failure.

**Electronic supplementary material:**

The online version of this article (doi:10.1186/s13054-014-0626-6) contains supplementary material, which is available to authorized users.

## Introduction

Correction of arterial hypotension is essential for adequate cellular metabolism [1,2]. Although there is no single mean arterial pressure (MAP) value that guarantees a global perfusion pressure [3], maintaining MAP above a minimum level has been recommended in order to prevent further tissue hypoperfusion and organ dysfunction [4-6]. In this regard, fluid administration is still considered the first-choice therapy to restore arterial pressure in most hemodynamic resuscitation protocols [4-6]. However, because arterial pressure results from the interaction between the arterial system and the blood ejected by the heart [7], the response of blood pressure to fluids continues to be a challenge [8-12]. Therefore, even if a patient is able to increase cardiac output (CO) with fluids, the arterial pressure response cannot easily be predicted [13]. So, in order to determine whether fluid administration will improve arterial pressure, it is necessary to evaluate not only patients’ preload dependency but also their arterial load [14]—that is, the net force imposed on left ventricular ejection that defines, along with left ventricular stroke volume (SV), the arterial pressure [15].

In a previous study, we found that dynamic arterial elastance (Ea_dyn_), defined as the pulse pressure variation (PPV) to stroke volume variation (SVV) ratio, could predict the arterial pressure increase after volume expansion (VE) in hypotensive, preload-dependent patients [13]. However, because both SVV and PPV were obtained from the pulse pressure analysis, mathematical coupling could not be rejected as a reason for the findings; thus, a validation study was necessary before Ea_dyn_ could be recommended for clinical decision-making [16].

The aim of this study was to confirm the usefulness of Ea_dyn_ as a predictor of the arterial pressure response to fluid administration by simultaneously measuring SVV and PPV from two independent signals.

## Methods

This observational study was conducted in the Intensive Care Unit of the Hospital SAS de Jerez during a 1-year period (from July 2012 to July 2013). Approval from our Institutional Research Ethics Committee (Cómite de Ética de la Investigación de Jerez-Sierra-Costa Noroeste, Acta 3, April 2012) was obtained. Informed consent was deemed unnecessary because the study protocol and the monitoring procedures were considered to be part of routine clinical care.

### Patients

We prospectively included all patients equipped with an indwelling arterial catheter and evaluated by esophageal Doppler monitoring who were receiving a fluid challenge for the presence of clinical signs of acute circulatory failure, including hypotension (defined as a MAP ≤65 mmHg or a systolic arterial pressure (SAP) ≤90 mmHg); requirement for vasopressor drugs, presence of lactic acidosis, urine output ≤0.5 ml∙kg^−1^∙hr^−1^ during at least 2 hours, heart rate >100 beats/min and/or the presence of skin mottling. Preload dependency was assessed according to our institutional protocol for hemodynamic resuscitation and defined as a CO increase ≥10% after a 2-minute leg-raising maneuver [17]. In all cases, the final decision to start or continue fluid administration was made by the treating physician.

Patients were under controlled mechanical ventilation with no spontaneous respiratory efforts, as assessed by visual inspection of the airway pressure curve. Patients with unstable cardiac rhythm were excluded, although this condition did not affect the decision to administer fluids.

### Hemodynamic monitoring

Patients were monitored with an esophageal Doppler monitoring system (CardioQ-Combi™; Deltex Medical, Chichester, UK). This system combines a standard Doppler monitor with arterial pressure analysis capability. The probe was inserted into the esophagus, preferably using the nasal route, and advanced until the maximal peak velocity of the aortic blood flow was reached. The gain setting was adjusted to obtain the optimum outline of the Doppler waveform. In order to reduce signal noise from heart valves and wall thump artifact, a built-in filter function was activated in some patients and kept unchanged throughout the study.

An arterial pressure transducer (TruWave; Edwards Lifesciences, Irvine, CA, USA) was zeroed to atmospheric pressure, and optimal damping of the arterial waveform was carefully checked by fast-flushing the line. The arterial pressure signal was transferred from the patient’s bedside monitor to the Doppler system using a serial cable and automatically synchronized with the aortic blood flow waveform for analysis (Additional file 1: Figure S1).

### Arterial load assessment

**Figure 1:**
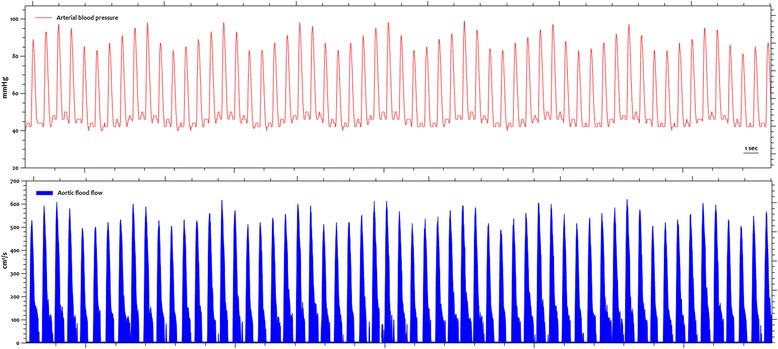
**An illustrative example of the arterial pressure and aortic blood flow recordings is shown.** Both signals are integrated into the esophageal Doppler system for analysis: the arterial pressure waveform from the patient’s bedside monitor and the aortic blood flow from the Doppler probe. Dynamic arterial elastance was calculated as the ratio between pulse pressure variation and stroke volume variation. All variables are automatically calculated by the Doppler monitor, which combines the arterial pressure analysis with the usual aortic blood flow measurements.

Our arterial load assessment was grounded on a basic physiological framework based on a two-element Windkessel model of arterial circulation with static and dynamic components. The static component represents a unique pressure–volume (P–V) relationship and can be described by a resistive element or the systemic vascular resistance (SVR = MAP/CO*80) and a pulsatile element or the net arterial compliance (C = SV/arterial pulse pressure) [18]. This distinction is a product of the oscillatory nature of the arterial flow and the mechanical properties of the arterial system [7]. The effective arterial elastance (Ea = 0.9*SAP/SV) is considered an integrative variable of arterial load that incorporates both steady and pulsatile elements [15,19,20]. Dynamic assessment of arterial load was performed by calculating Ea_dyn_, which we defined as the ratio between PPV and SVV during a respiratory cycle. Rather than being a steady-state variable, Ea_dyn_ depicts the slope of the P–V relationship and provides a functional assessment of arterial load [21]. PPV obtained from the arterial line and SVV from the aortic blood flow were simultaneously calculated by using the Doppler monitor software with the standard formulae [22] and averaged over three respiratory cycles (Figure 1). In order to do so, respiratory rate was manually introduced on the Doppler monitor.

### Study protocol

All hemodynamic variables were measured prior to and just after VE, consisting of 500 ml of normal saline given within 30 minutes. No changes in ventilatory settings or vasoactive drugs were made during volume administration. No paralyzing agents were used for the study. All hemodynamic variables were automatically recorded every 10 seconds and averaged over 1 minute for statistical purposes.

### Statistical analysis

The normality of the data distribution was tested using the Kolmogorov-Smirnov test. The results are expressed as mean ± standard deviation or as median (25th to 75th interquartile range), as appropriate. Patients were classified as hypotensive if they had a MAP ≤65 mmHg and/or SAP <90 mmHg. Sepsis was defined according to standard criteria [5]. As we selected an increase in CO ≥10% for defining fluid responsiveness, we chose a similar cutoff for MAP changes for defining a positive pressure response. This threshold was selected assuming a matched ventriculoarterial coupling and optimal hydraulic efficiency (maximal stroke work) [23]. The relationships between variables were analyzed using a linear regression analysis. Because changes in arterial pressure depend not only on Ea_dyn_ but also on the magnitude of CO changes, we also calculated the weighted least-squares regression, taking into consideration the contribution of CO changes in the relationship between arterial pressure increase and preinfusion Ea_dyn_. Differences at baseline between pressure responders and non-responders were compared by means of an independent sample *t*-test, and their evolution over time was assessed by one-way analysis of variance with repeated measurements, using group (pressure responders vs. non-responders) as the between-subjects factor and time (preinfusion vs. postinfusion) as the within-subjects factor. Differences between groups were compared using an independent samples *t*-test. Comparison between preinfusion and postinfusion periods was tested using a *t*-test for repeated measurements. Categorical variables were compared using the χ^2^ test. For each arterial load variable, a receiver operating characteristic (ROC) curve was created to test the ability of predicting a positive MAP response after VE. Areas under the ROC curve (AUC) with 95% confidence intervals (95% CIs) were compared by using the method described by DeLong *et al.* [24]. Optimal cutoff values were calculated by maximizing the Youden index (J = sensitivity + specificity −1). The diagnostic performance of Ea_dyn_ was also assessed on the basis of its positive and negative likelihood ratios (LHRs). A diagnostic variable with potential to influence clinical decisions is usually considered when its positive LHR is >10, its negative LHR is <0.1 and the AUC is >0.9 [25]. A gray zone for Ea_dyn_ cutoff was created using a resampling method [26]. In summary, the Youden index for each bootstrapped sample from 1,000 replications of the original study population was calculated, then the median value and the 95% CI of these 1,000 optimal cut-offs were obtained. This bootstrapped 95% CI defines a gray zone around the optimum criterion in which formal conclusions about prediction of MAP response cannot be obtained [25,26].

A preliminary power analysis determined that a sample size of 48 patients was required for detecting an AUC difference of 0.1 (α = 0.05; β = 0.20; allocation ratio = 1:1).

A *P*-value <0.05 was considered significant. All statistical analyses were two-tailed and performed using MedCalc statistical software version 14.8.0 [27].

## Results

The patients’ characteristics are summarized in Table 1. A total of 80 VEs performed in 53 patients were studied (median, 1; interquartile range (IQR): 1 to 2; maximum: 3 per patient). Eight VEs were excluded from the analysis because CO did not increase by ≥10%, and one was excluded because of the presence of cardiac arrhythmia during recoding. The hemodynamic profiles of these eight non-preload responder patients are shown in Additional file 1: Table S1. In seven patients (13%), the arterial blood pressure was monitored from a femoral arterial catheter. Thirty-two patients (60%) had sepsis, mainly from an abdominal source. The patients were studied mostly during the first 24 hours of ICU admission. The heart rate/respiratory rate ratio was 4.9 ± 0.9 at preinfusion and 4.7 ± 0.9 after VE.

**Table 1:** **Characteristics and demographic data** ^**a**^

**Demographics**	**Data**
Age (yr)	62.7 ± 14.4
Sex (men/women)	31/22
Weight (kg)	81 ± 23
Height (cm)	167 ± 8
APACHE II score at admission	21 ± 5
Plasma lactate level at admission (mmol/L)	1.9 (1.21 to 3.12)
ICU mortality rate, *n* (%)	16 (30%)
Vasoactive drugs at time of inclusion	
Norepinephrine, *n*; dose (μg kg^−1^ min^−1^)	30; 0.19 ± 0.14
Dobutamine, *n*; dose (μg kg^−1^ min^−1^)	13; 5 ± 2
Analgesia and sedative drugs	
Fentanyl, *n*; dose (μg kg^−1^ hr^−1^)	28; 1.55 ± 0.57
Remifentanil, *n*; dose (μg kg^−1^ min^−1^)	20; 0.14 ± 0.06
Midazolam, *n*; dose (mg kg^−1^ hr^−1^)	32; 0.10 ± 0.04
Propofol, *n*; dose (mg kg^−1^ hr^−1^)	3; 1.25 (1 to 2)
Morphine, *n*; dose (mg kg^−1^ hr^−1^)	1; 1.8
Ventilator settings	
Tidal volume (ml/kg predicted body weight)	8 (6 to 10)
Respiratory rate (breaths/min)	19 (18 to 20)
Total PEEP (cmH_2_O)	8 (6 to 10)
Acute circulatory failure origin, *n* (%)	
Postoperative hypovolemia	7 (13%)
Hemorrhagic shock	4 (8%)
Anoxic encephalopathy	2 (4%)
Toxic poisoning	2 (4%)
Sepsis/septic shock	32 (60%)
Abdominal	18
Pulmonary	8
Urological	2
Neurological	3
Other	1

^a^Values are expressed as mean ± SD, median (25th to 75th percentile) or absolute numbers, as appropriate. APACHE II, Acute Physiology and Chronic Health Evaluation II; ICU: intensive care unit; PEEP: positive end-expiratory pressure.

### Hemodynamic response to volume expansion

**Figure 2:**
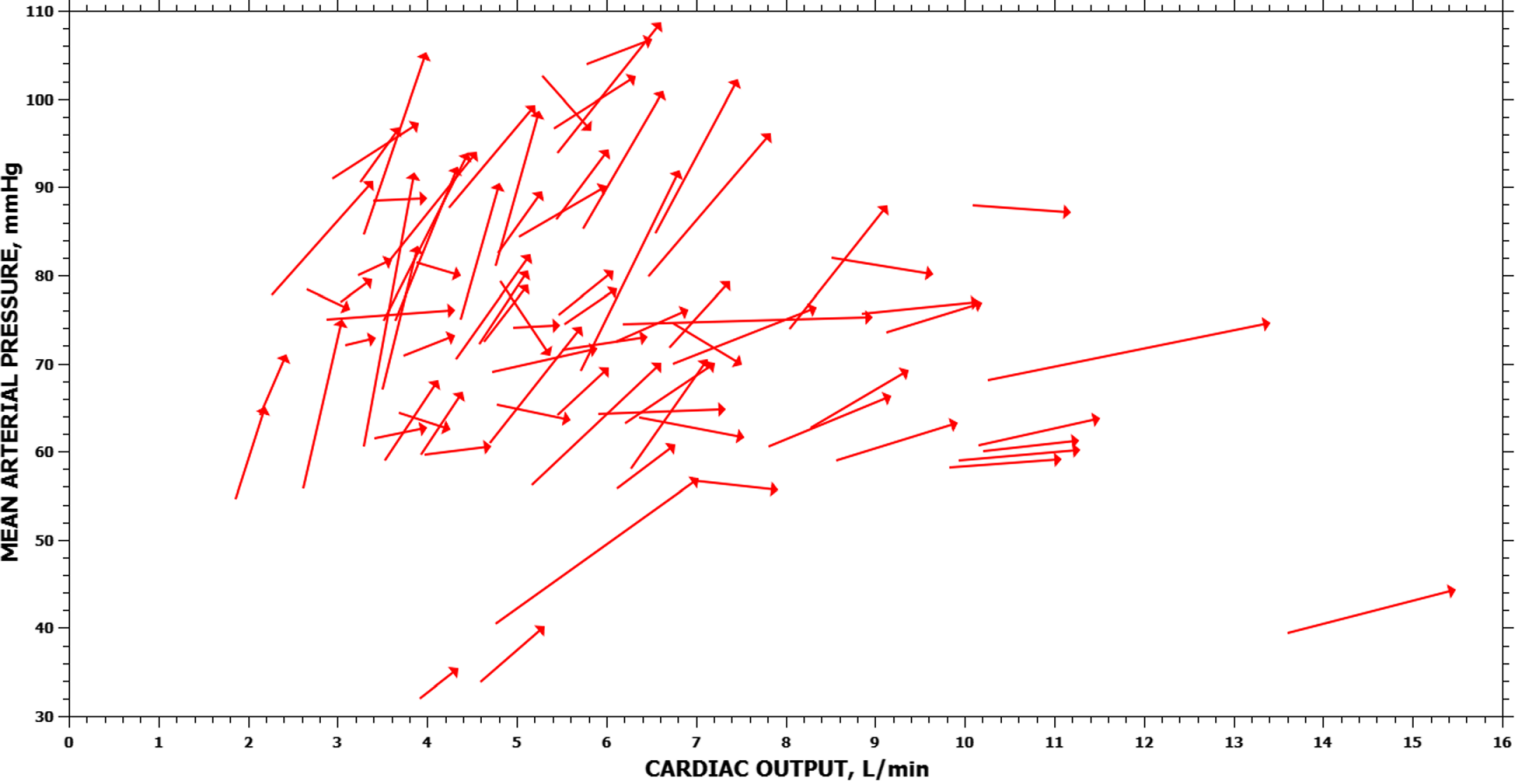
**Individual changes in cardiac output and mean arterial pressure after fluid administration.**

Hemodynamic changes after VE are shown in Table 2. Overall, VE increased CO by 14.7% (13.2% to 19.1%), SV by 20.1% (17.1% to 21.9%) and MAP by 9.8% (4.9 to 10.3%). Thirty-three patients (41%) were classified as pressure responders. Of 32 fluid challenges performed in the 22 hypotensive patients, only 17 showed a MAP increase by ≥10% (53%). The rate of pressure responders was similar between patients with and without sepsis (34% vs. 51%; *P* = 0.18), or between hypotensive and non-hypotensive patients (53% vs. 33%; *P* = 0.13). There was a weak relationship between VE-induced changes in MAP and CO (*R*^2^ = 0.05; *P* = 0.04) (Figure 2) and between VE-induced changes in arterial pulse pressure and SV (*R*^2^ = 0.13; *P* <0.001).

**Table 2:** **Effects of volume expansion in hemodynamic variables according to mean arterial pressure increase** ^**a**^

	**Before volume expansion**	**After volume expansion**	***P***-**value** ^**b**^
CO, L/min			
Responders	4.9 ± 2.2	5.8 ± 2.5^c^	0.581
Non-responders	5.9 ± 2.3	6.8 ± 2.6^c^	
Heart rate, beats/min			
Responders	91 ± 21	86 ± 19^c^	0.036
Non-responders	92 ± 17	90 ± 17^c^	
SV, ml			
Responders	56 ± 24	69 ± 26^c^	0.971
Non-responders	65 ± 27	77 ± 33^c^	
CPO, W			
Responders	0.7 ± 0.3^d^	1.0 ± 0.4^c^	<0.001
Non-responders	0.9 ± 0.3	1.1 ± 0.4^c^	
MAP, mmHg			
Responders	67 ± 15^d^	80 ± 18^c^	<0.001
Non-responders	74 ± 12	76 ± 12^c^	
SAP, mmHg			
Responders	102 ± 18^d^	128 ± 22^c^	<0.001
Non-responders	113 ± 18	118 ± 20^*^	
DAP, mmHg			
Responders	51 ± 13	57 ± 14^c^	<0.001
Non-responders	55 ± 11	55 ± 11	
PP, mmHg			
Responders	51 ± 17	70 ± 20^c^	<0.001
Non-responders	58 ± 15	64 ± 17^c^	
PPV, %			
Responders	18 ± 7^d^	9 ± 5^c^	<0.001
Non-responders	11 ± 5	8 ± 4^c^	
SVV, %			
Responders	17 ± 8	15 ± 7^c^	0.135
Non-responders	18 ± 7	15 ± 5^c^	

^a^Responders are defined by a mean arterial pressure (MAP) increase ≥10%). Data are expressed as mean ± SD. ^b^ *P*-values refer to group (responder vs. non-responder) and time (preinfusion vs. postinfusion) interaction using analysis of variance for repeated measurements. ^c^ *P* <0.05 vs. before volume expansion. ^†^ *P* <0.05 vs. non-responders. CO, Cardiac output; CPO, Cardiac power output (mean arterial pressure × cardiac output/451); DAP, Diastolic arterial pressure; MAP, Mean arterial pressure; PP, Pulse pressure (systolic pressure minus diastolic pressure); PPV, Arterial pulse pressure variation; SAP, Systolic arterial pressure; SV, Stroke volume; SVV, Stroke volume variation.

### Effects of volume expansion on arterial load

Fluid administration decreased Ea from 1.75 (1.54 to 1.91) mmHg/ml to 1.59 (1.45 to 1.81) mmHg/ml (*P* <0.0001) and SVR from 1,229 ± 554 dyn∙s∙cm^−5^ to 1,150 ± 514 dyn∙s∙cm^−5^ (*P* <0.0001). Net arterial compliance did not change. In non-responder patients, VE reduced the static component of arterial load but did not affect Ea_dyn_ (Table 3).

**Table 3:** **Effects of fluid administration on static and dynamic arterial load variables according to mean arterial pressure increase** ^**a**^

	**Before volume expansion**	**After volume expansion**	***P***-**value** ^**b**^
Ea_dyn_			
Responders	1.04 ± 0.28^c^	0.62 ± 0.27^d^	<0.001
Non-responders	0.60 ± 0.14	0.59 ± 0.23	
Ea, mmHg/ml			
Responders	1.89 ± 0.77	1.89 ± 0.68	<0.001
Non-responders	1.82 ± 0.76	1.58 ± 0.62^d^	
C, ml/mmHg			
Responders	1.11 ± 0.36	0.99 ± 0.34^d^	<0.001
Non-responders	1.17 ± 0.57	1.27 ± 0.60^d^	
SVR, dyn∙s∙cm^−5^			
Responders	1282 ± 572	1293 ± 548	<0.001
Non-responders	1192 ± 545	1050 ± 469^d^	

^a^Responders were defined as mean arterial pressure increase ≥10% after fluid administration. Data are expressed as mean ± SD. ^b^ *P*-values refer to group (responders vs. non-responders) and time (preinfusion vs. postinfusion) interaction using analysis of variance for repeated measurements. ^c^ *P* <0.0001 vs. non-responders. ^d^ *P* <0.0001 vs. before volume expansion. C, Net arterial compliance; Ea, Effective arterial elastance; Ea_dyn_, Dynamic arterial elastance; SVR, Systemic vascular resistance.

**Figure 3:**
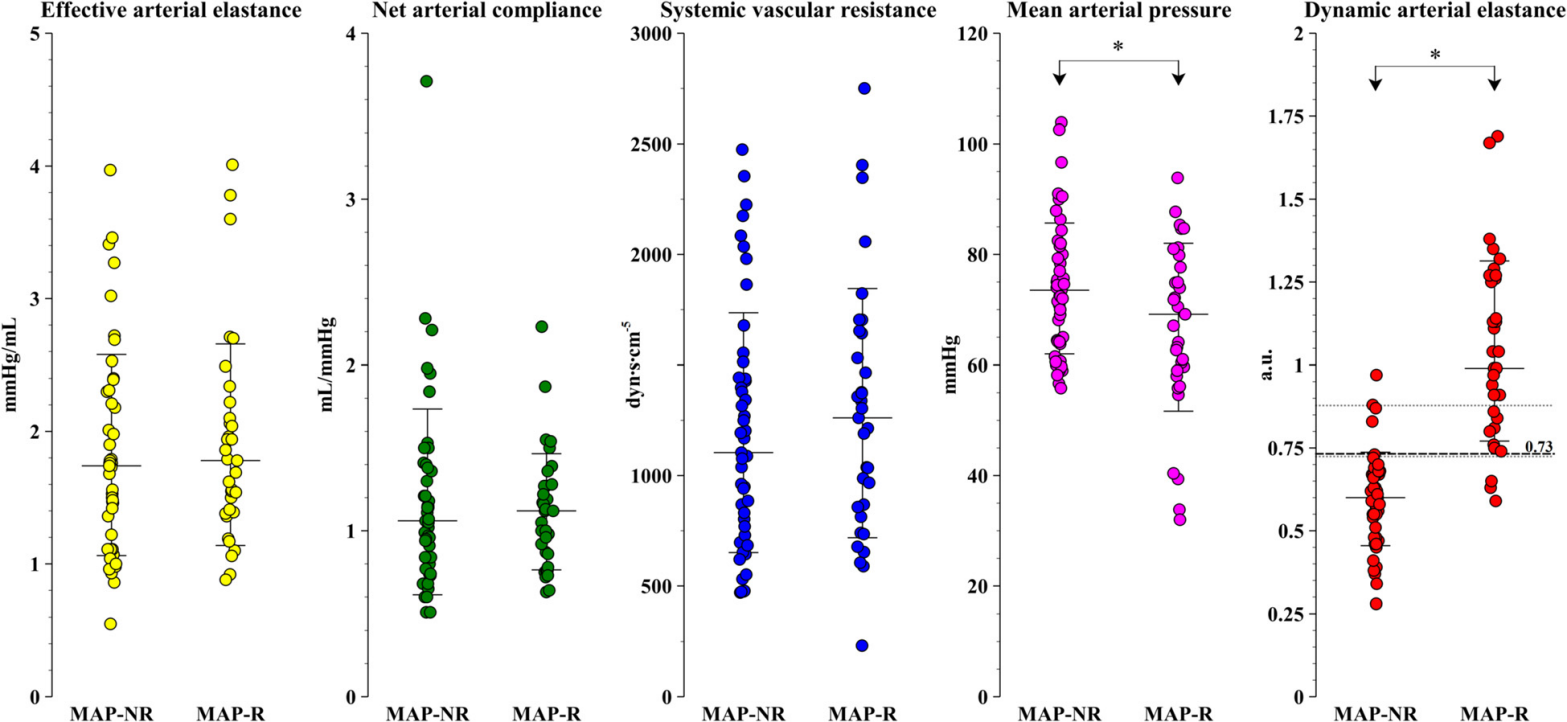
**Distribution of individual values (open circles) and mean ± SD (lines) of arterial load variables before fluid administration in pressure responders.** The dashed line represents the optimal cutoff for dynamic arterial elastance corresponding to maximum Youden index, and dotted lines depict the “gray zone” calculated from bootstrapped 95% confidence interval (0.72 to 0.88). MAP-R, Mean arterial pressure responders defined by increase ≥10%; MAP-NR, Mean arterial pressure non-responders defined by increase <10%. *P <0.0001 for MAP-R vs. MAP-NR.

At baseline, Ea_dyn_ was higher in pressure responder patients (1.04 ± 0.28 vs. 0.60 + 0.14; *P* <0.0001) (Figure 3). Pressure responders were also more hypotensive (Table 2). Furthermore, preinfusion Ea_dyn_ and SVR were higher in hypotensive patients (Ea_dyn_: 0.90 ± 0.35 vs. 0.70 ± 0.23, *P* <0.01; SVR: 1,036 ± 605 dyn∙s∙cm^−5^ vs. 1,359 ± 486 dyn∙s∙cm^−5^, *P* <0.05) (Additional file 1: Table S2). No differences were observed in preinfusion arterial load variables between septic and non-septic patients (Additional file 1: Table S3).

**Figure 4:**
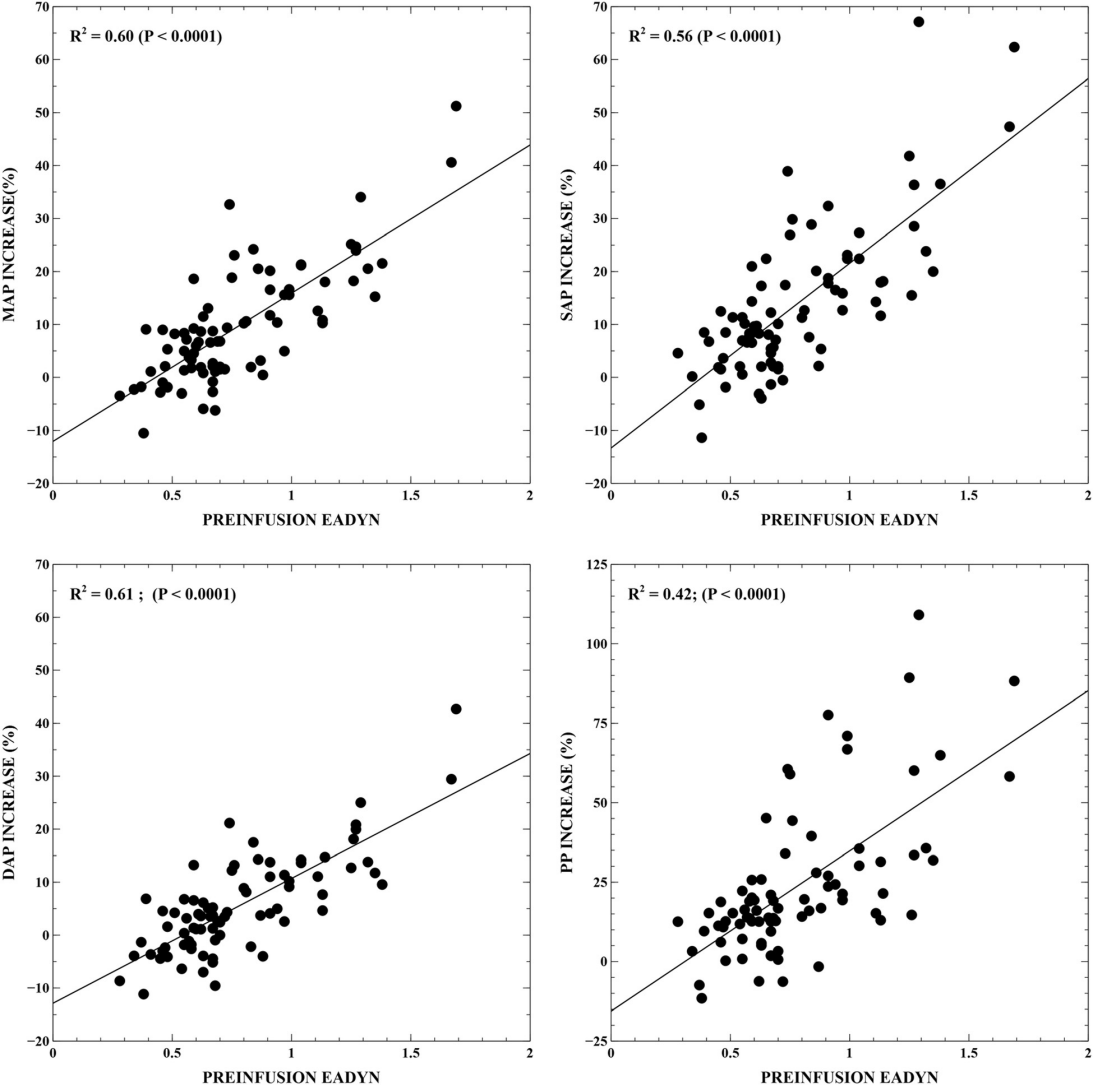
**Linear regression analysis of the relationship between preinfusion dynamic arterial elastance (Ea**_**dyn**_ **) and changes in arterial pressure induced by fluid administration.** DAP: diastolic arterial pressure; MAP: mean arterial pressure; SAP: systolic arterial pressure; PP: arterial pulse pressure.

Preinfusion Ea_dyn_ was correlated with VE-induced changes in SAP (*R*^2^ = 0.56; *P* <0.0001), diastolic arterial pressure (DAP; *R*^2^ = 0.61; *P* <0.0001), MAP (*R*^2^ = 0.60; *P* <0.0001) and arterial pulse pressure (*R*^2^ = 0.42; *P* <0.0001) (Figure 4). When adjusted for CO changes, this relationship was higher (SAP: *R*^2^ = 0.62; DAP: *R*^2^ = 0.70; MAP: *R*^2^ = 0.69; pulse pressure: *R*^2^ = 0.44; *P* <0.0001, respectively; Additional file 1: Figures S2 and S3). None of the static components of arterial load were associated with changes in arterial pressure produced by VE. No relationship was observed between Ea_dyn_ and other variables of arterial load at baseline or after VE.

### Prediction of arterial pressure increase after volume expansion

**Figure 5:**
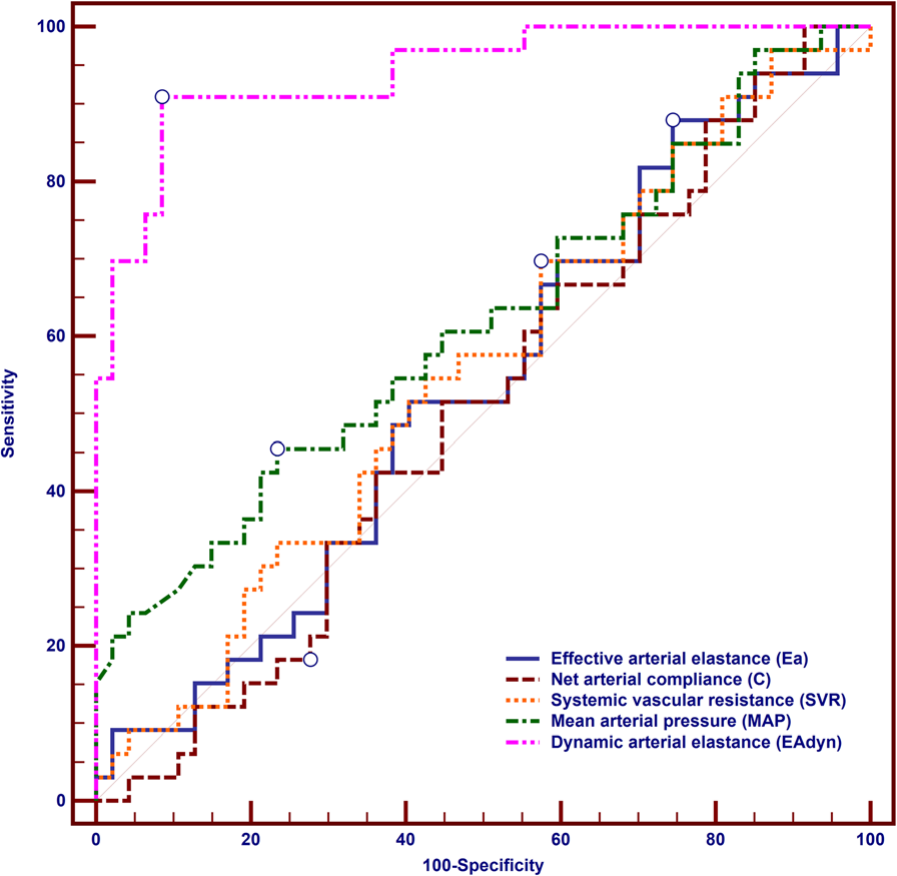
**Comparison of receiver operating characteristic curves for testing the ability of static and dynamic arterial load variables to detect a mean arterial pressure increase ≥10% after volume administration.** Dynamic arterial elastance (Ea_dyn_) = area under the receiver operating characteristic curve (AUC): 0.94 (95% CI: 0.86 to 0.98); effective arterial elastance (Ea) = AUC: 0.53 (95% CI: 0.42 to 0.65); systemic vascular resistance (SVR) = AUC: 0.55 (95% CI: 0.44 to 0.66); net arterial compliance (C) = AUC: 0.51 (95% CI: 0.39 to 0.62); preinfusion MAP = AUC: 0.62 (95% CI: 0.50 to 0.72).

The AUC for preinfusion Ea_dyn_ (0.94 ± 0.03; 95% CI: 0.86 to 0.98) was higher than for any other variable of arterial load: Ea (0.53 ± 0.07; 95% CI: 0.42 to 0.65; *P* <0.0001), C (0.51 ± 0.07; 95% CI: 0.39 to 0.62; *P* <0.0001); SVR (0.55 ± 0.07; 95% CI: 0.44 to 0.66; *P* <0.0001) and preinfusion MAP (0.62 ± 0.07; 95% CI: 0.50 to 0.72; *P* <0.0001) (Figure 5).

At baseline, an Ea_dyn_ value ≥0.73 predicted an increase ≥10% in MAP after VE with a sensitivity of 90.9% (95% CI: 75.7% to 98.1%) and a specificity of 91.5% (95% CI: 79.6% to 97.6%), with a positive predictive value of 88.2 (95% CI: 72.5% to 96.7%) and a negative predictive value of 93.5 (95% CI: 82.1% to 98.6%). The positive and negative LHRs for Ea_dyn_ were 10.68 (95% CI: 4.2 to 27.4) and 0.1 (95% CI: 0.03 to 0.3), respectively. The bootstrapped 95% CI defined a gray zone for Ea_dyn_ ranging between 0.72 and 0.88. Only ten fluid challenges (12.5%) were situated in the inconclusive zone.

When only the first VE per patient was included in the analysis, the predictive performance of Ea_dyn_ was similar (AUC: 0.92 ± 0.04; 95% CI: 0.81 to 0.98; *P* = 0.64 vs. including all fluid challenges) (Additional file 1: Figure S4). When we considered an increase of CO and MAP ≥15% for defining a positive preload and pressure response, the AUC of Ea_dyn_ was also excellent: 0.97 ± 0.03 (95% CI: 0.86 to 0.99; *P* = 0.37 vs. definition of ≥10% for CO and MAP increases) (Additional file 1: Figure S5). Because the impact of PPV seems to be predominant on the Ea_dyn_ ratio, we also compared the performance of PPV alone against Ea_dyn_ (AUC for PPV: 0.76; 95% CI: 0.65 to 0.85; *P* <0.001 vs. AUC for Ea_dyn_) (Additional file 1: Figure S6). The ability of Ea_dyn_ for predicting pressure responsiveness was similar between hypotensive and non-hypotensive patients, as well as between septic and non-septic patients (Additional file 1: Tables S4 and S5).

## Discussion

In this study, we confirmed the ability of Ea_dyn_ for predicting arterial pressure response to VE in preload-dependent patients with acute circulatory failure. The greatest strength of our findings is that both SVV and PPV were simultaneously obtained from two independent signals: esophageal Doppler monitor–derived aortic blood flow and the arterial waveform from a routine arterial line. So, mathematical coupling can be excluded. Moreover, the predictive performance of Ea_dyn_ was similar between septic and non-septic patients and regardless of the presence of systemic hypotension.

Maintaining a constant perfusion pressure against a variable CO is a hallmark function of an efficient cardiovascular system [23,28]. Arterial pressure can be considered as a complex interface between the blood ejected by the heart, which is modulated to meet the metabolic demands of the organism, and the arterial vascular tree, an adaptive system influenced by its physical proprieties, neurohormonal factors and baroreflex function [29]. Arterial hypotension, therefore, is the pathological consequence of loss of balance between these determinants and often represents the first sign of an acute decompensated cardiovascular system [23,28].

When hypotension occurs, fluid administration remains as the first recommended therapy for restoring arterial pressure in current hemodynamic resuscitation protocols [4-6]. However, the assumption that increasing CO will be followed by an increase to the same extent in blood pressure is not often true [8-12], and vasopressors are frequently introduced when an arbitrary amount of fluids has been administered [4,5]. So, as long as no physiological trigger for the timing of vasopressor support is established, aggressive volume resuscitation, if aimed to a pressure target, could lead to fluid overload, delayed vasoactive therapy or even increased mortality risk [30].

On the basis of the functional hemodynamic concept [31], Ea_dyn_ was originally conceived to answer an eminently clinical question: If a patient is fluid-responsive, will arterial pressure improve with volume administration [14]? Taking advantage of well-known hemodynamic effects of intermittent positive pressure ventilation on left ventricular SV and arterial pressure [32], Ea_dyn_ depicts the slope of the P–V curve during a respiratory cycle, allowing the assessment of pressure responsiveness or, put another way, defining the flow dependency of arterial pressure. Consequently, if Ea_dyn_ is high and the patient is preload-dependent, arterial pressure will improve along with CO after VE. On the contrary, if Ea_dyn_ is low, even if the patient is fluid-responsive, VE will not increase blood pressure and vasopressors should be considered in order to correct hypotension. Moreover, for the same increase in CO, the greater the preinfusion Ea_dyn_, the greater the improvement in arterial pressure after VE. In our study, an Ea_dyn_ value of 0.73 predicted pressure responsiveness with a high sensitivity and specificity and a narrow gray zone of uncertainty. Interestingly, this threshold is close to the value found in our previous study [13] and similar to that suggested in the original publication [14].

It is noteworthy that Ea_dyn_ should not be interpreted as an actual variable of arterial load or arterial tone, but as a functional measure of arterial load. Therefore, in the same way that neither PPV nor SVV is an index of cardiac preload, Ea_dyn_ should not be considered a direct measure of arterial load. The lack of relationship observed in this study between static variables and Ea_dyn_ supports this hypothesis.

In our previous study, we observed that none of the static arterial load variables predicted the subsequent arterial pressure response to VE [13]. Our presently reported results confirm this observation in a larger group of patients with a different methodology. Although the lack of prediction of static variables of arterial load could be explained by several hypotheses, one reason that might be argued is that our arterial load framework, although physiologically reasonable, provides only a gross oversimplification of the actual nature of arterial system that ignores, for example, the phenomenon of pressure wave reflection [29]. A fuller characterization of arterial load requires a frequency domain assessment using technology outside the scope of routine hemodynamic monitoring [15,19]. However, we think that another explanation could be that the P–V relationship is not constant, because it can be also affected by age, sex or pathological conditions, such us septic shock or arterial hypertension [33]. So, even in the same patient, a single steady-state P–V relationship could be associated to different slopes and hence different Ea_dyn_ values.

Our study has some limitations that need to be mentioned. First, we decided to use the esophageal Doppler monitor for estimating CO and SVV. This is a well-validated method with proved benefits for guiding fluid therapy during the perioperative period [34]. However, because this technique assumes a constant proportion of CO through the descending aorta and a fixed aortic diameter, changes in flow distribution to the upper part of the body or variations in aortic diameter induced by arterial pressure changes could potentially affect its capability to detect CO changes [35]. Moreover, the ability of esophageal Doppler monitoring for tracking beat-to-beat changes in SV, as may occur during an inferior vena cava occlusion, has raised some concerns about the accuracy of this method for calculating SVV [36,37]. More recently, two clinical studies have demonstrated the usefulness of SVV measured by esophageal Doppler monitoring for predicting fluid responsiveness in surgical patients [38,39]. Despite these limitations, we chose esophageal Doppler monitoring because of its ability to detect rapid hemodynamic changes and its low dependency on arterial tone variations, unlike pulse pressure analysis-based systems [40]. Specifically, our monitor combines the standard Doppler method with the arterial pressure analysis, allowing the simultaneous assessment of both flow and pressure, and the automatic calculation of arterial load variables.

A second limitation is that we included preload-dependent patients, regardless of their baseline MAP levels. Obviously, measuring Ea_dyn_ should be considered primarily in hypotensive patients. However, we think that knowing whether arterial pressure will improve with fluids could also be of interest for the clinician, because some patients could benefit from fluids administered in order to decrease their vasopressor dosage [41]. Finally, given that a predefined level of MAP does not guarantee an adequate perfusion pressure to all tissues and cannot be generalized to all patients, hemodynamic resuscitation should be targeted not only at restoring perfusion pressure but also at providing a sufficient oxygen delivery to guarantee an adequate cellular metabolism [2]. However, correction of arterial hypotension seems to be a necessary condition for normal cellular function [1].

## Conclusions

Ea_dyn_ obtained from two independent signals allowed the prediction of arterial pressure response to fluid administration in mechanically ventilated, preload-dependent patients with acute circulatory failure. The clinical applicability of Ea_dyn_ seems now purely limited by technological boundaries. Only its implementation in future hemodynamic resuscitation protocols will determine the impact of Ea_dyn_ in patient outcome.

## Key messages

- Dynamic arterial elastance (Ea_dyn_), defined as the ratio between pulse pressure variation (PPV) and stroke volume variation (SVV), measured from two independent signals, predicted the arterial pressure response to fluid administration in mechanically ventilated, preload-dependent patients.
- An Ea_dyn_ value ≥0.73 predicted a mean arterial pressure increase ≥10% after volume expansion with 90.9% sensitivity and 91.5% specificity, as well as a narrow gray zone ranging between 0.72 and 0.88. The predictive performance of Ea_dyn_ was similar in septic and non-septic patients and regardless of the presence of systemic hypotension.
- The arterial pressure response to volume administration was not related to static arterial load variables.
- Preload-dependent patients in whom arterial pressure did not increase after volume expansion showed a decrease in the static component of arterial load.

## Additional file

Additional file 1:
**Supplementary information and data.**

## Abbreviations

AUC: Area under the receiver operating characteristic curve
C: Net arterial compliance
CI: Confidence interval
CO: Cardiac output
DAP: Diastolic arterial pressure
Ea: Effective arterial elastance
Ea_dyn_: Dynamic arterial elastance
ICU: Intensive care unit
IQR: Interquartile range
LHR: Likelihood ratio
MAP: Mean arterial pressure
ROC: Receiver operating characteristic
SAP: Systolic arterial pressure
SD: Standard deviation
SV: Stroke volume
SVR: Systemic vascular resistance
VE: Volume expansion

## References

[1] Vincent JL, De Backer D (2013). Circulatory shock. N Engl J Med.

[2] Pinsky MR (2000). Both perfusion pressure and flow are essential for adequate resuscitation. Sepsis.

[3] LeDoux D, Astiz ME, Carpati CM, Rackow EC (2000). Effects of perfusion pressure on tissue perfusion in septic shock. Crit Care Med.

[4] Antonelli M, Levy M, Andrews PJ, Chastre J, Hudson LD, Manthous C, Meduri GU, Moreno RP, Putensen C, Stewart T, Torres A (2007). Hemodynamic monitoring in shock and implications for management. International Consensus Conference, Paris, France, 27–28 April 2006. Intensive Care Med.

[5] Dellinger RP, Levy MM, Carlet JM, Bion J, Parker MM, Jaeschke R, Reinhart K, Angus DC, Brun-Buisson C, Beale R, Calandra T, Dhainaut JF, Gerlach H, Harvey M, Marini JJ, Marshall J, Ranieri M, Ramsay G, Sevransky J, Thompson BT, Townsend S, Vender JS, Zimmerman JL, Vincent JL (2008). Surviving Sepsis Campaign: international guidelines for management of severe sepsis and septic shock: 2008. Intensive Care Med.

[6] Ochagavía A, Baigorri F, Mesquida J, Ayuela JM, Ferrándiz A, García X, Monge MI, Mateu L, Sabatier C, Clau-Terré F, Vicho R, Zapata L, Maynar J, Gil A, Grupo de Trabajo de Cuidados Intensivos Cardiológicos y RCP de la SEMICYUC (2014). [Hemodynamic monitoring in the critically patient: recommendations of the Cardiological Intensive Care and CPR Working Group of the Spanish Society of Intensive Care and Coronary Units] [Article in Spanish]. Med Intensiva.

[7] Nichols WW, O’Rourke M: **The nature of flow of a liquid.** In *McDonald’s Blood Flow in Arteries: Theoretical, Experimental and Clinical Principles.* 5th edition. Edited by Nichols WW, O’Rourke M. London: Oxford University Press; 2005:11–48.

[8] Pierrakos C, Velissaris D, Scolletta S, Heenen S, De Backer D, Vincent JL (2012). Can changes in arterial pressure be used to detect changes in cardiac index during fluid challenge in patients with septic shock?. Intensive Care Med.

[9] Dufour N, Chemla D, Teboul JL, Monnet X, Richard C, Osman D (2011). Changes in pulse pressure following fluid loading: a comparison between aortic root (non-invasive tonometry) and femoral artery (invasive recordings). Intensive Care Med.

[10] Lakhal K, Ehrmann S, Perrotin D, Wolff M, Boulain T (2013). Fluid challenge: tracking changes in cardiac output with blood pressure monitoring (invasive or non-invasive). Intensive Care Med.

[11] Monnet X, Letierce A, Hamzaoui O, Chemla D, Anguel N, Osman D, Richard C, Teboul JL (2011). Arterial pressure allows monitoring the changes in cardiac output induced by volume expansion but not by norepinephrine. Crit Care Med.

[12] Le Manach Y, Hofer CK, Lehot JJ, Vallet B, Goarin JP, Tavernier B, Cannesson M (2012). Can changes in arterial pressure be used to detect changes in cardiac output during volume expansion in the perioperative period?. Anesthesiology.

[13] Monge García MI, Gil Cano A, Gracia Romero M (2011). Dynamic arterial elastance to predict arterial pressure response to volume loading in preload-dependent patients. Crit Care.

[14] Pinsky MR: **Protocolized cardiovascular management based on ventricular-arterial coupling.** In *Functional Hemodynamic Monitoring.* Edited by Pinsky MR, Payen D. Berlin: Springer-Verlag; 2006:381–395.

[15] Sunagawa K, Maughan WL, Burkhoff D, Sagawa K (1983). Left ventricular interaction with arterial load studied in isolated canine ventricle. Am J Physiol.

[16] Pinsky MR (2011). Defining the boundaries of bedside pulse contour analysis: dynamic arterial elastance. Crit Care.

[17] Monnet X, Teboul JL (2013). Assessment of volume responsiveness during mechanical ventilation: recent advances. Crit Care.

[18] Chemla D, Hébert JL, Coirault C, Zamani K, Suard I, Colin P, Lecarpentier Y (1998). Total arterial compliance estimated by stroke volume-to-aortic pulse pressure ratio in humans. Am J Physiol.

[19] Kelly RP, Ting CT, Yang TM, Liu CP, Maughan WL, Chang MS, Kass DA (1992). Effective arterial elastance as index of arterial vascular load in humans. Circulation.

[20] Segers P, Stergiopulos N, Westerhof N (2002). Relation of effective arterial elastance to arterial system properties. Am J Physiol Heart Circ Physiol.

[21] Pinsky MR: **Functional hemodynamic monitoring: applied physiology at the bedside.** In *Yearbook of Intensive Care and Emergency Medicine.* Edited by Vincent JL. Heidelberg: Springer-Verlag; 2002:534–551.

[22] Michard F, Teboul JL (2000). Using heart-lung interactions to assess fluid responsiveness during mechanical ventilation. Crit Care.

[23] Guarracino F, Baldassarri R, Pinsky MR (2013). Ventriculo-arterial decoupling in acutely altered hemodynamic states. Crit Care.

[24] DeLong ER, DeLong DM, Clarke-Pearson DL (1988). Comparing the areas under two or more correlated receiver operating characteristic curves: a nonparametric approach. Biometrics.

[25] Ray P, Le Manach Y, Riou B, Houle TT (2010). Statistical evaluation of a biomarker. Anesthesiology.

[26] Cannesson M, Le Manach Y, Hofer CK, Goarin JP, Lehot JJ, Vallet B, Tavernier B (2011). Assessing the diagnostic accuracy of pulse pressure variations for the prediction of fluid responsiveness: a “gray zone” approach. Anesthesiology.

[27] MedCalc Statistical Software, MedCalc Software bvba, Ostend, Belgium; 2014. [http://www.medcalc.org]

[28] Lamia B, Chemla D, Richard C, Teboul JL (2005). Clinical review: interpretation of arterial pressure wave in shock states. Crit Care.

[29] Nichols WW, O’Rourke M (2005). McDonald’s Blood Flow in Arteries: Theoretical, Experimental and Clinical principles.

[30] Beck V, Chateau D, Bryson GL, Pisipati A, Zanotti S, Parrillo JE, Kumar A (2014). Timing of vasopressor initiation and mortality in septic shock: a cohort study. Crit Care.

[31] Garcia X, Pinsky MR (2011). Clinical applicability of functional hemodynamic monitoring. Ann Intensive Care.

[32] Pinsky MR (2012). Heart lung interactions during mechanical ventilation. Curr Opin Crit Care.

[33] Nichols WW, O’Rourke M: **Contours of pressure and flow waves in arteries.** In *McDonald’s Blood Flow in Arteries: Theoretical, Experimental and Clinical principles.* 5th edition. Edited by Nichols WW, O’Rourke M. London: Oxford University Press; 2005:165–191.

[34] Peyton PJ, Chong SW (2010). Minimally invasive measurement of cardiac output during surgery and critical care: a meta-analysis of accuracy and precision. Anesthesiology.

[35] Monnet X, Chemla D, Osman D, Anguel N, Richard C, Pinsky MR, Teboul JL (2007). Measuring aortic diameter improves accuracy of esophageal Doppler in assessing fluid responsiveness. Crit Care Med.

[36] Gunn SR, Kim HK, Harrigan PW, Pinsky MR (2006). Ability of pulse contour and esophageal Doppler to estimate rapid changes in stroke volume. Intensive Care Med.

[37] Marquez J, McCurry K, Severyn DA, Pinsky MR (2008). Ability of pulse power, esophageal Doppler, and arterial pulse pressure to estimate rapid changes in stroke volume in humans. Crit Care Med.

[38] Guinot PG, de Broca B, Abou Arab O, Diouf M, Badoux L, Bernard E, Lorne E, Dupont H (2013). Ability of stroke volume variation measured by oesophageal Doppler monitoring to predict fluid responsiveness during surgery. Br J Anaesth.

[39] Guinot PG, de Broca B, Bernard E, Abou Arab O, Lorne E, Dupont H (2014). Respiratory stroke volume variation assessed by oesophageal Doppler monitoring predicts fluid responsiveness during laparoscopy. Br J Anaesth.

[40] Monge Garcia MI, Gracia Romero M, Gil Cano A, Rhodes A, Grounds RM, Cecconi M (2013). Impact of arterial load on the agreement between pulse pressure analysis and esophageal Doppler. Crit Care.

[41] Vos JJ, Kalmar AF, Struys MM, Wietasch JK, Hendriks HG, Scheeren TW (2013). Comparison of arterial pressure and plethysmographic waveform-based dynamic preload variables in assessing fluid responsiveness and dynamic arterial tone in patients undergoing major hepatic resection. Br J Anaesth.

