# Supplementary material for: Dynamic arterial elastance as a predictor of arterial pressure response to fluid administration: a validation study
Source: Crit Care. 2014 Nov 19;18(6):626. doi: 10.1186/s13054-014-0626-6 (PMC4271484; doi:10.1186/s13054-014-0626-6)
Supplement: Additional file 1: — Supplementary information and data. [file 13054_2014_626_MOESM1_ESM.docx]

**Dynamic arterial elastance as a predictor of arterial pressure changes after volume administration: a validation study**

Manuel Ignacio Monge García^1,2^, Manuel Gracia Romero^1^, Anselmo Gil Cano^1^, Hollmann D. Aya^2^, Andrew Rhodes^2^, Robert Michael Grounds^2^, Maurizio Cecconi^2^

^1^Servicio de Cuidados Intensivos y Urgencias, Hospital SAS de Jerez, C/ Circunvalación s/n, 11407 Jerez de la Frontera, Spain

^2^Department of Intensive Care Medicine, St. George’s Healthcare NHS Trust and St George’s University of London, Tooting, London SW17 0QT, UK

**ELECTRONIC SUPLEMENTARY FILE (ESM)**


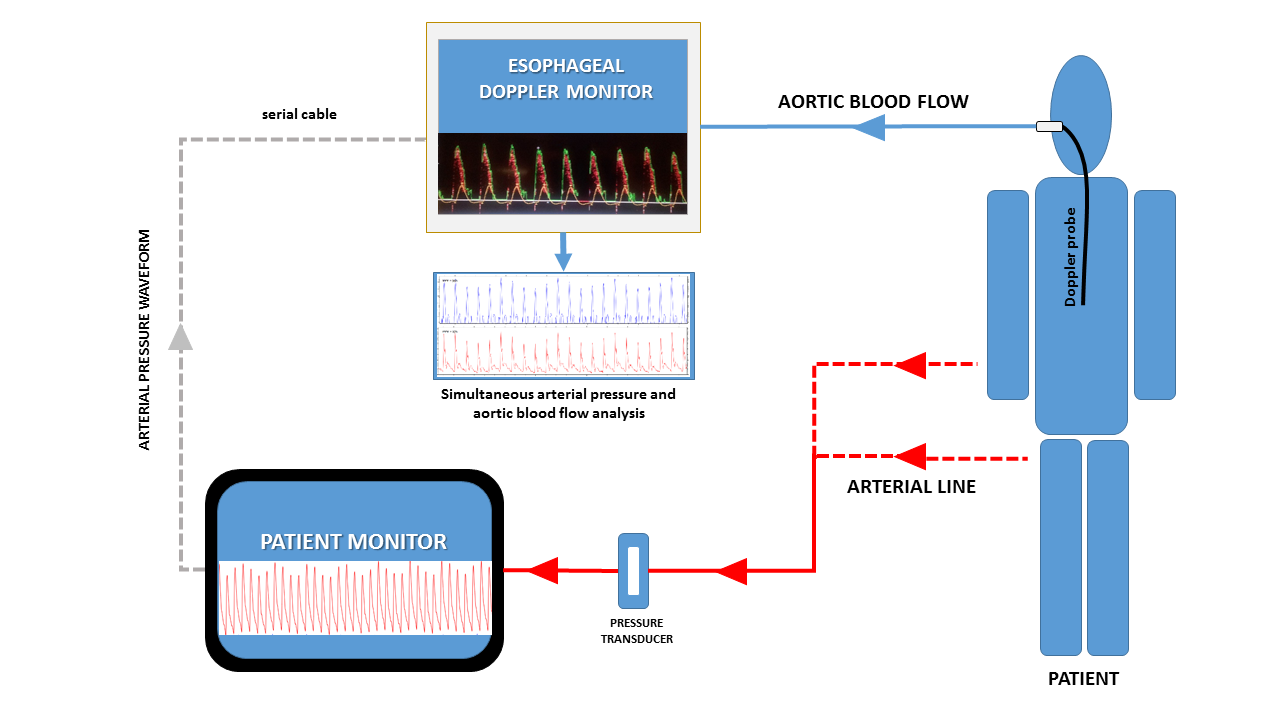


**Figure S1. Schematic representation of the monitoring procedures.**

| **Figure S2. Weighted least-squares regression analysis between preinfusion dynamic arterial elastance and arterial pressure increases after volume expansion, considering relative changes in cardiac output.** | |
| --- | --- |
| 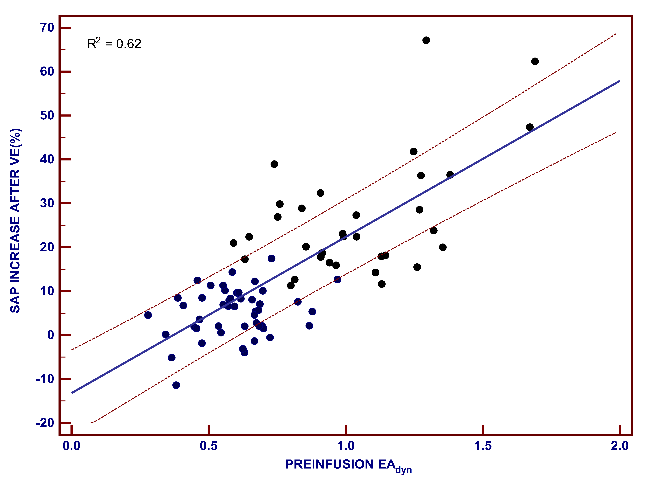 | 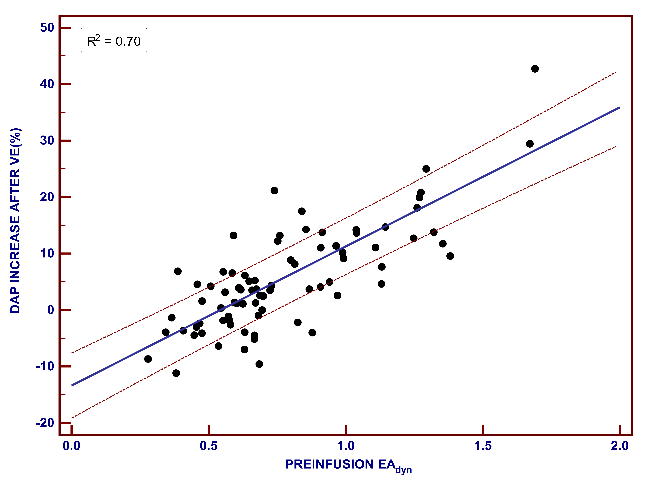 |
| 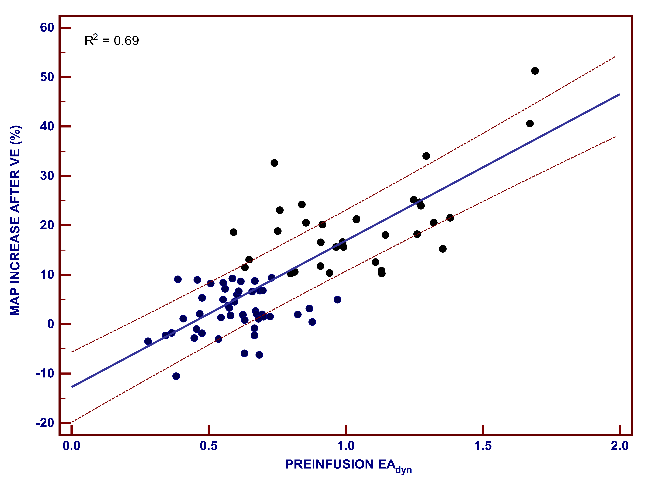 | 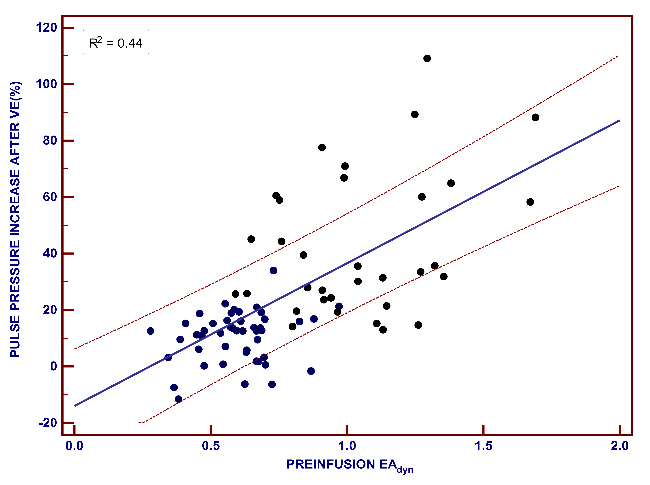 |
| Relationship between preinfusion dynamic arterial elastance (Ea_dyn_) and systolic arterial pressure (SAP), diastolic arterial pressure (DAP), mean arterial pressure (MAP) and arterial pulse pressure changes after volume expansion (VE), adjusted for VE-induced cardiac output changes. Dashed lines represent 95% confidence intervals for the regression line (solid line). | |

| **Figure S3. Relationship between cardiac output and arterial pressure changes after volume expansion and preinfusion dynamic arterial elastance.** |
| --- |
| 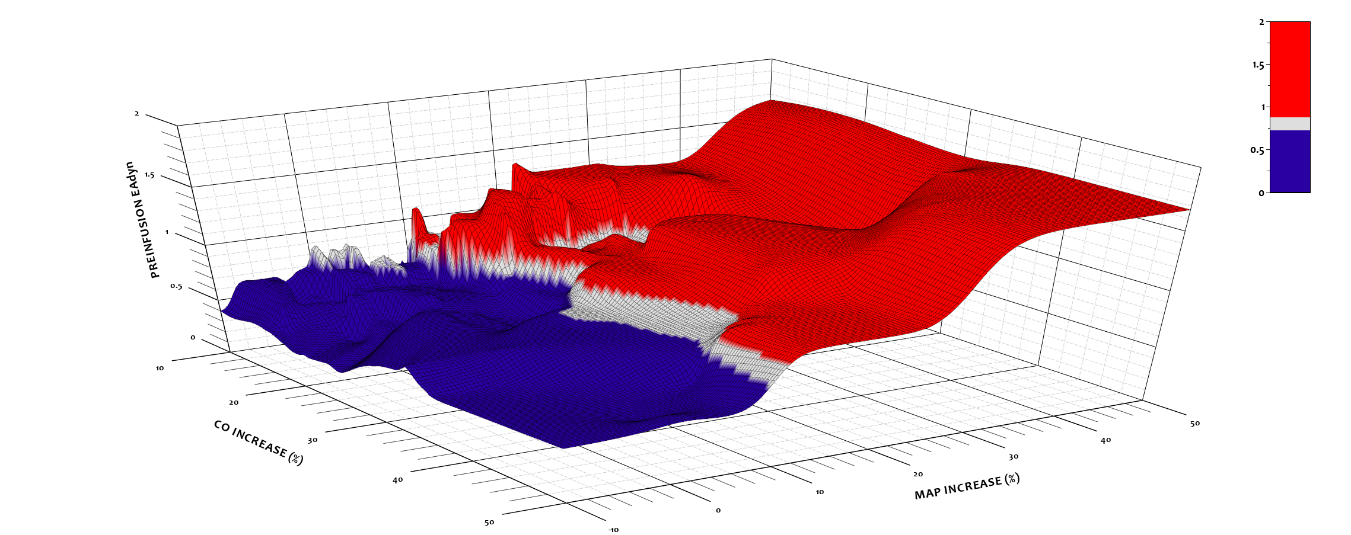 |
| 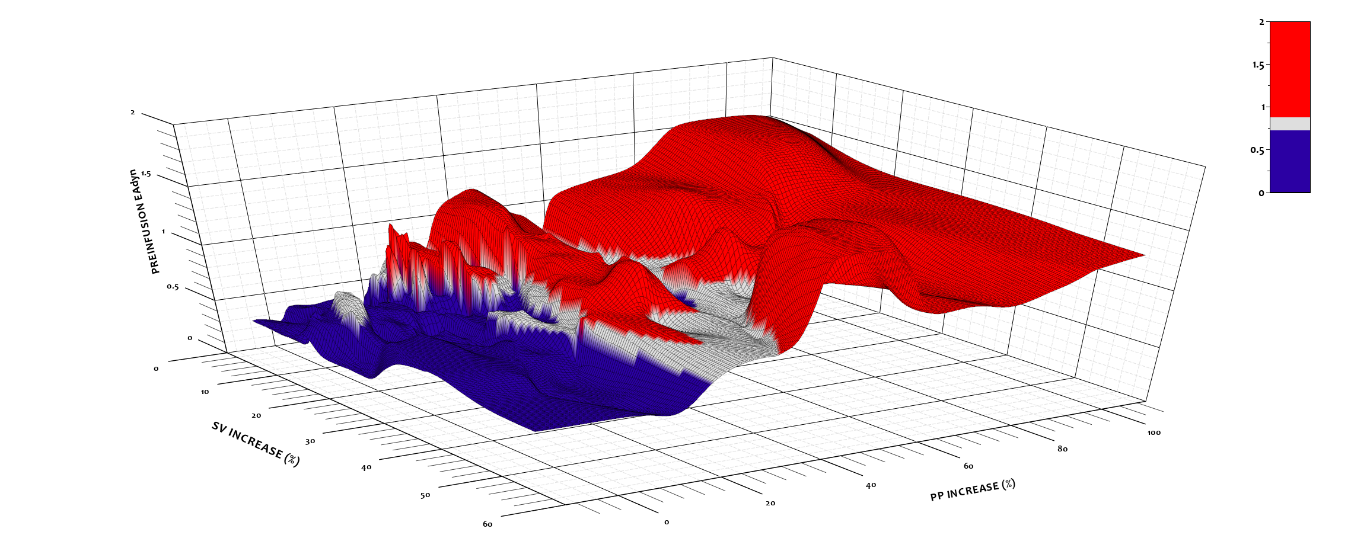 |
| Cardiac output (CO) and mean arterial pressure (MAP) increases after volume expansion (VE) and preinfusion dynamic arterial elastance (Ea_dyn_) relationship (above), and stroke volume (SV) and arterial pulse pressure (PP) increases after VE and preinfusion Ea_dyn_ relationship (below). Colors are defined according to preinfusion Ea_dyn_ value: < 0.72 in blue, from 0.72 to 0.88 (gray zone) in gray; and > 0.88 in red. |

| **Figure S4. Predictive performance of arterial load parameters for predicting an increase ≥ 10% in MAP after fluid administration, when only the first fluid challenge per patient was considered (n = 53; pressure-responders = 22 patients).** | | |
| --- | --- | --- |
| 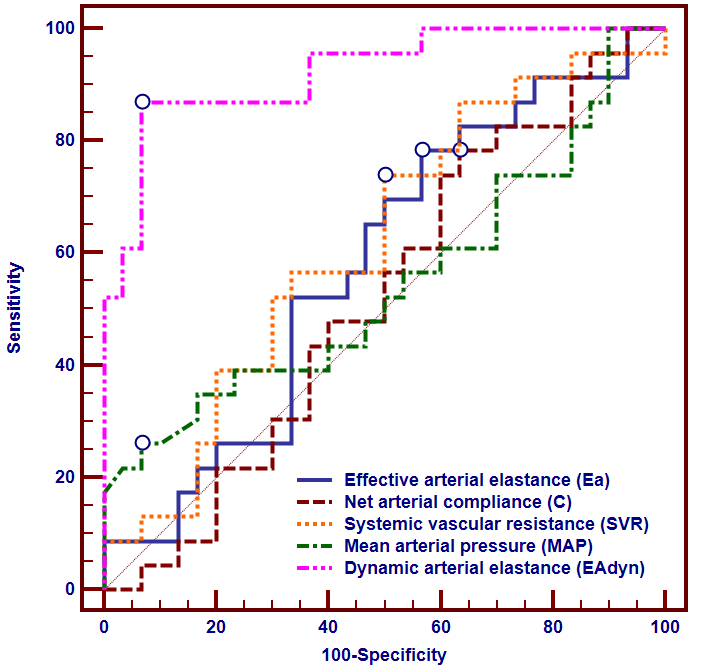 | | |
|  | **AUC (95%CI)** | **SE** |
| **Ea_dyn_** | 0.92 (0.81 – 0.98)^*^ | 0.04 |
| **Ea, mmHg/mL** | 0.58 (0.44 – 0.71) | 0.08 |
| **C, mL/mmHg** | 0.52 (0.38 – 0.66) | 0.08 |
| **SVR, dyn∙s∙cm^-5^** | 0.62 (0.47 – 0.75) | 0.08 |
| **MAP, mmHg** | 0.55 (0.40 – 0.68) | 0.08 |
| AUC: area under the receiver operating curve; C: net arterial compliance; Ea: effective arterial elastance; Ea_dyn_: dynamic arterial elastance; MAP: mean arterial pressure; SE: standard error; SVR: systemic vascular resistance.  *The area under the ROC curve for Ea_dyn_ was significantly higher than for other arterial load parameters (p < 0.001) according to DeLong test. | | |

| **Figure S5. Predictive performance of arterial load parameters for predicting an increase ≥ 15% in MAP in patients with a cardiac output increase ≥ 15% after fluid administration (n=39; pressure-responders = 18 patients).** | | |
| --- | --- | --- |
| 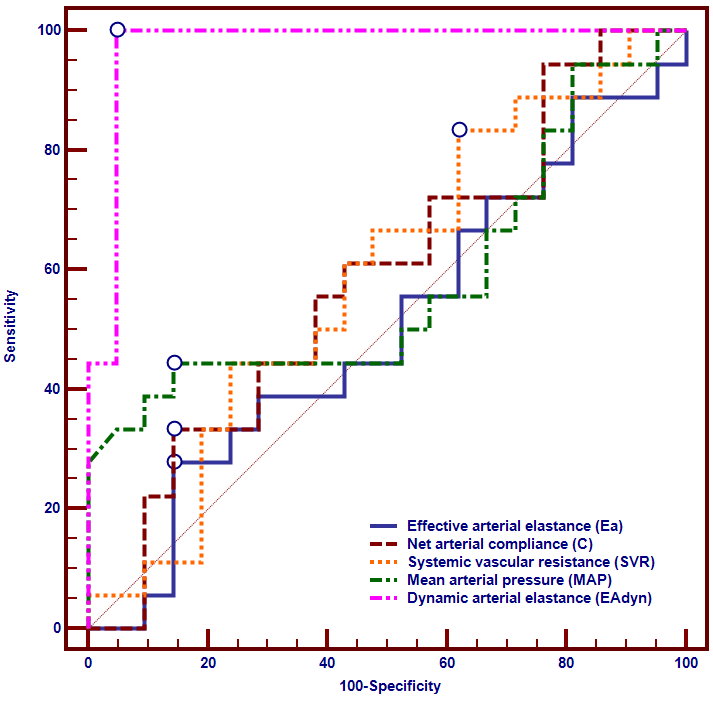 | | |
|  | **AUC (95%CI)** | **SE** |
| **Ea_dyn_** | 0.97 (0.86 – 0.99)^*^ | 0.03 |
| **Ea, mmHg/Ml** | 0.50 (0.34 – 0.67) | 0.10 |
| **C, mL/mmHg** | 0.58 (0.42 – 0.74) | 0.09 |
| **SVR, dyn∙s∙cm^-5^** | 0.59 (0.42 – 0.70) | 0.09 |
| **MAP, mmHg** | 0.58 (0.41 – 0.74) | 0.10 |
| AUC: area under the receiver operating curve; C: net arterial compliance; Ea: effective arterial elastance; Ea_dyn_: dynamic arterial elastance; MAP: mean arterial pressure; SE: standard error; SVR: systemic vascular resistance.  *The area under the ROC curve for Ea_dyn_ was significantly higher than for other arterial load parameters (p < 0.001) according to DeLong test. | | |

| **Figure S6. Comparison of ROC curves for testing the ability of pulse pressure variation (PPV) and dynamic arterial elastance (Ea_dyn_) to detect a mean arterial pressure (MAP) increase ≥ 10% after volume administration** | | |
| --- | --- | --- |
| 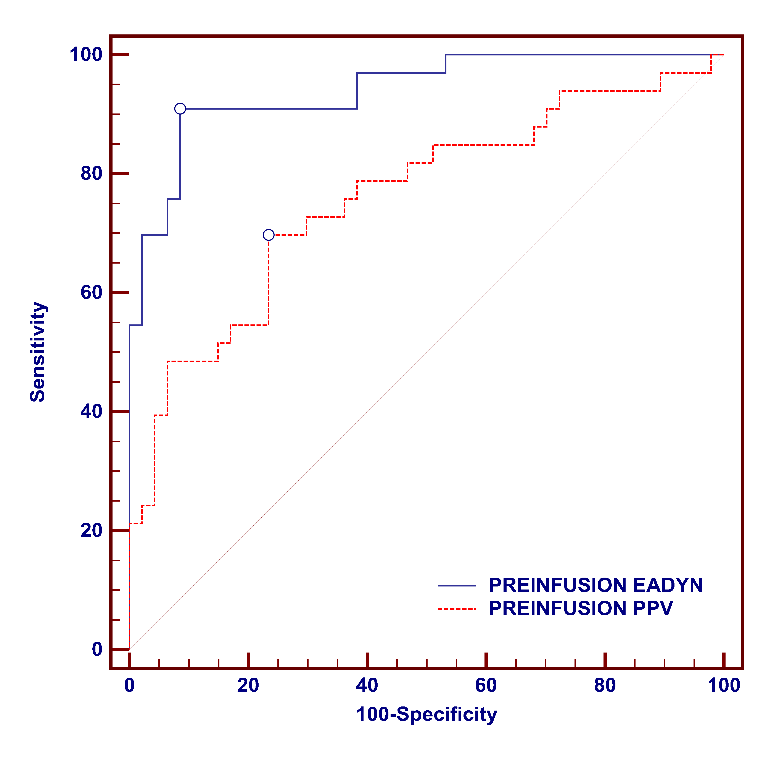 | | |
|  | **AUC (95%CI)** | **SE** |
| **Ea_dyn_** | 0.94 (0.86 – 0.98)^*^ | 0.03 |
| **PPV, %** | 0.76 (0.65 – 0.85) | 0.06 |
| AUC: area under the receiver operating curve; Ea_dyn_: dynamic arterial elastance; PPV: pulse pressure variation; SE: standard error.  *The area under the ROC curve for Ea_dyn_ was significantly higher than for PPV (p < 0.001) according to DeLong test. | | |

| **Table 1. Effects of volume expansion (VE) in the 8 non-preload responder patients (CO increase < 10%) excluded from the analysis.** | | |
| --- | --- | --- |
|  | **Before VE** | **After VE** |
| **CO, L/min** | 5.14 (3.49 to 6.02) | 5.31 (3.64 to 6.14)^*^ |
| **HR, beats/min** | 80 (69 to 86) | 74 (66 to 80)^*^ |
| **SV, mL** | 60 (46 to 105) | 70 (50 to 104)^*^ |
| **CPO, W** | 0.84 (0.56 to 1.10) | 0.92 (0.58 to 1.10)^*^ |
| **MAP, mmHg** | 71 (66 to 74) | 72 (68 to 78) |
| **SAP, mmHg** | 114 (107 to 118) | 116 (111 to 125) |
| **DAP, mmHg** | 52 (45 to 56) | 52 (46 to 56) |
| **PP, mmHg** | 59 (47 to 72) | 61 (51 to 82)^*^ |
| **PPV, %** | 12 (8 to 15) | 8 (5 to 11)^*^ |
| **SVV, %** | 16 (9 to 20) | 13 (9 to 16) |
| **Ea_dyn_** | 0.67 (0.65 to 0.82) | 0.64 (0.49 to 0.77) |
| **Ea, mmHg/mL** | 1.64 (1.01 to 2.22) | 1.45 (1.10 to 2.07) |
| **C, mL/mmHg** | 1.21 (0.86 to 1.46) | 1.24 (0.91 to 1.31) |
| **SVR, dyn∙s∙cm^-5^** | 1346 (925 to 1649) | 1278 (980 to 1579) |
| Data are expressed as median (interquartile range). CO: cardiac output; CPO: cardiac power output; DAP: diastolic arterial pressure; Ea: effective arterial elastance; Ea_dyn_: dynamic arterial elastance; HR: heart rate; MAP: mean arterial pressure; PP: pulse pressure (systolic minus diastolic pressure); PPV: pulse pressure variation; SAP: systolic arterial pressure; SV: stroke volume; SVR: systemic vascular resistance; SVV: stroke volume variation.  * p < 0.05 vs. before volume expansion (Wilcoxon test). | | |

| **Table S2. Preinfusion arterial load parameters comparison between hypotensive and non-hypotensive patients.** | | | |
| --- | --- | --- | --- |
|  | **Hypotensive**  **(n = 32)** | **Non-hypotensive**  **(n = 48)** | ***p*** |
| **Ea_dyn_** | 0.90 ± 0.35 | 0.70 ± 0.24 | < 0.01 |
| **Ea, mmHg/mL** | 1.73 ± 0.81 | 1.93 ± 0.73 | 0.26 |
| **C, mL/mmHg** | 1.16 ± 0.41 | 1.15 ± 0.54 | 0.93 |
| **SVR, dyn∙s∙cm^-5^** | 1036 ± 605 | 1359 ± 486 | < 0.05 |
| Data are expressed as mean ± SD. C: net arterial compliance; Ea: effective arterial elastance; Ea_dyn_: dynamic arterial elastance; SVR: systemic vascular resistance. | | | |

| **Table S3. Preinfusion arterial load parameters comparison between septic and non-septic patients.** | | | |
| --- | --- | --- | --- |
|  | **Septic**  **(n = 48)** | **Non-septic**  **(n = 34)** | ***p*** |
| **Ea_dyn_** | 0.78 ± 0.29 | 0.81 ± 0.33 | 0.62 |
| **Ea, mmHg/mL** | 1.80 ± 0.73 | 1.93 ± 0.81 | 0.45 |
| **C, mL/mmHg** | 1.18± 0.53 | 1.13 ± 0.43 | 0.66 |
| **SVR, dyn∙s∙cm^-5^** | 1143 ± 535 | 1360 ± 573 | 0.08 |
| Data are expressed as mean ± SD. C: net arterial compliance; Ea: effective arterial elastance; Ea_dyn_: dynamic arterial elastance; SVR: systemic vascular resistance. | | | |

| **Table S4. Comparison of areas under the ROC curve for predicting arterial pressure response (MAP increase ≥ 10%) after volume expansion between hypotensive and non-hypotensive patients.** | | | |
| --- | --- | --- | --- |
|  | **Hypotensive**  **(n = 32)** | **Non-hypotensive**  **(n = 48)** | ***p*** |
| **Ea_dyn_** | 0.98 (0.85 – 1) | 0.90 (0.78 – 0.97) | 0.15 |
| **Ea, mmHg/mL** | 0.66 (0.47 – 0.82) | 0.51 (0.37 – 0.66) | 0.28 |
| **C, mL/mmHg** | 0.63 (0.44 – 0.79) | 0.60 (0.45 – 0.74) | 0.81 |
| **SVR, dyn∙s∙cm^-5^** | 0.69 (0.51 – 0.84) | 0.50 (0.35 – 0.65) | 0.13 |
| **MAP, mmHg** | 0.65 (0.46 – 0.81) | 0.51 (0.37 – 0.66) | 0.32 |
| Data are expressed as AUC (95%CI). C: net arterial compliance; Ea: effective arterial elastance; Ea_dyn_: dynamic arterial elastance; MAP: mean arterial pressure; SVR: systemic vascular resistance. | | | |

| **Table S5. Comparison of areas under the ROC curve for predicting arterial pressure response (MAP ≥ 10%) after VE between septic and non-septic patients** | | | |
| --- | --- | --- | --- |
|  | **Septic**  **(n = 48)** | **Non-septic**  **(n = 34)** | ***p*** |
| **Ea_dyn_** | 0.92 (0.81 – 0.98) | 0.96 (0.83 – 0.99) | 0.41 |
| **Ea, mmHg/mL** | 0.53 (0.38 – 0.67) | 0.50 (0.33 – 0.68) | 0.86 |
| **C, mL/mmHg** | 0.54 (0.39 – 0.69) | 0.53 (0.35 – 0.70) | 0.92 |
| **SVR, dyn∙s∙cm^-5^** | 0.52 (0.37 – 0.66) | 0.53 (0.35 – 0.70) | 0.95 |
| **MAP, mmHg** | 0.58 (0.42 – 0.72) | 0.65 (0.46 – 0.80) | 0.59 |
| Data are expressed as AUC (95%CI). C: net arterial compliance; Ea: effective arterial elastance; Ea_dyn_: dynamic arterial elastance; MAP: mean arterial pressure; SVR: systemic vascular resistance. | | | |
